# In vitro propagation, carotenoid, fatty acid and tocopherol content of *Ajuga multiflora* Bunge

**DOI:** 10.1007/s13205-016-0376-z

**Published:** 2016-03-14

**Authors:** Iyyakkannu Sivanesan, Ramesh Kumar Saini, Rafi Noorzai, Ahmad Jawid Zamany, Doo Hwan Kim

**Affiliations:** 1 Department of Molecular Biotechnology, Konkuk University, 1, Hwayang-dong, Gwangjin-gu, Seoul, 143-701 South Korea; 2 Department of Bio-resources and Food Science, Konkuk University, Seoul, 143-701 South Korea

**Keywords:** *Ajuga multiflora*, 6-Benzyladenine, Bioactive compounds, Shoot proliferation, Shoot tip, Thidiazuron

## Abstract

The effect of plant growth regulators on shoot proliferation from shoot tip explants of *Ajuga multiflora* was studied. The highest number of shoots (17.1) was observed when shoot tip explants were cultured on Murashige and Skoog (MS) medium fortified with 8.0 µM 6-Benzyladenine (BA) and 2.7 µM α-naphthaleneacetic acid (NAA). The mean number of shoots per explant was increased 1.6-fold in liquid medium as compared with semi-solid medium. Maximum rooting (100 %) with an average of 7.2 roots per shoot was obtained on MS basal medium. Rooted plantlets were successfully acclimatised in the greenhouse with 100 % survival rate. Composition of carotenoids, fatty acids and tocopherols was also studied from leaves of greenhouse-grown plants and in vitro-regenerated shoots of *A. multiflora*. The greatest amounts of carotenoids, fatty acids and tocopherols were obtained from leaves of in vitro-regenerated shoots cultured on MS basal medium, followed by leaves of greenhouse-grown plants and leaves of in vitro-regenerated shoots cultured on MS basal medium with 2.0 µM BA or thidiazuron. The most abundant carotenoid in *A. multiflora* leaves was all-E-lutein (89.4–382.6 μg g^−1^ FW) followed by all-E-β-carotene (32.0–156.7 μg g^−1^ FW), 9′-Z-neoxanthin (14.2–63.4 μg g^−1^ FW), all-E-violaxanthin (13.0–45.9 μg g^−1^ FW), all-E-zeaxanthin (1.3–2.5 μg g^−1^ FW) and all-E-β-cryptoxanthin (0.3–0.9 μg g^−1^ FW). α-Tocopherol was the predominant tocopherol in *A. multiflora* leaves. Linolenic acid (49.03–52.59 %) was detected in higher amounts in *A. multiflora* leaf samples followed by linoleic acid (18.95–21.39 %) and palmitic acid (15.79–18.66 %).

**Electronic supplementary material:**

The online version of this article (doi:10.1007/s13205-016-0376-z) contains supplementary material, which is available to authorized users.

## Introduction

The genus *Ajuga* (Lamiaceae) includes several ornamental and medicinal species distributed in the cooler parts of Asia, Africa, Australia and Europe. *Ajuga* species are used for the treatment of diabetes, diarrhoea, fever, gastrointestinal disorders and high blood pressure in traditional medicine. Phytochemical studies revealed that *Ajuga* species contains several bioactive compounds such as anthocyanidin-glucosides, essential oils, iridoid glycosides, flavonoids, phytoecdysteroids, sterols, terpenoids and withanolides (Israili and Lyoussi 2009). *Ajuga multiflora* Bunge is a perennial ornamental herb distributed in China, Korea, Siberia and Russia. It has been used for the treatment of fever in Korean folk medicine. *A. multiflora* is reported to contain a number of phytoecdysteroids, which have been shown to possess pesticidal activity against several insect pests (Chi et al. 2002). Owing to its medicinal importance, ornamental value and pesticidal activity, this plant has been overexploited. It is typically propagated by division of rhizomes, rooted cuttings or seeds. However, conventional propagation of *A. multiflora* is hampered by several factors such as poor seed viability, dependence on season and slow vegetative multiplication (Sivanesan et al. 2011). Thus, an effective large-scale propagation method is urgently needed to provide enough plant material for commercial exploitation (Sivanesan and Park 2015). Micropropagation is a useful method for mass clonal propagation of *A. multiflora*. Though in vitro propagation methods have been developed for *A. multiflora* (Sivanesan et al. 2011; Sivanesan and Jeong 2014; Sivanesan and Park 2015), there have been no published reports available on in vitro propagation of *A. multiflora* using shoot tip explants. Thus, the efficiency of shoot tip explants to regenerate *A. multiflora* plants has been explored.

Plant lipids are group of molecules that mainly include carotenoids, fatty acids, sterols and tocopherols. These molecules are essential for both human and plant health (Fiedor and Burda 2014). Lipophilic antioxidants such as carotenoids and tocopherols are frequently added to cosmetic, food and pharmaceutical products (Alvarez and Rodriguez 2000; Lu et al. 2015). Fatty acids are often prone to oxidation; thus, lipophilic antioxidants are found to co-exist with plant lipids, protecting the integrity and vitality of the plant (Tang et al. 2015). In vitro-developed calli, shoots and roots can be utilised to extract the phytochemicals (Jeong and Sivanesan 2015). Several bioactive compounds are produced by plant cell, tissue and organ cultures (Gandhi et al. 2015). Many studies on the production of valuable compounds such as anthocyanin, flavonoids, phenolics and phytoecdysteroids from in vitro cell, organ and hairy root cultures have been carried out on *Ajuga* species (Terahara et al. 1996; Callebaut et al. 1997; Madhavi et al. 1997; Kim et al. 2005; Cheng et al. 2008). However, no studies on the production of carotenoids, fatty acids and tocopherol from cell and organ cultures of *A. multiflora* have been reported. Thus, analysis of these compounds will provide a better understanding on biological activities of this plant species. The aims of this study were to (1) determine the effect of plant growth regulators (PGRs) on axillary shoot proliferation from shoot tip explants of *A. multiflora* and (2) evaluate the carotenoids, fatty acids and tocopherols contents in leaves of greenhouse-grown plants and in vitro-developed shoots.

## Materials and methods

### Axillary shoot proliferation

Actively growing shoots were collected from 6-month-old greenhouse-grown plants (Sivanesan and Park 2015). The shoots were washed under running tap water and then washed thoroughly in distilled water (DH_2_O). The remaining procedures were done in a sterile laminar airflow chamber. The explants were disinfected in a 70 % (v/v) ethanol solution for 60 s, 2.0 % (v/v) sodium hypochlorite for 10 min and 0.1 % (w/v) mercuric chloride for 10 min. Each treatment was followed by four washes with sterile DH_2_O. The culture medium consisted of Murashige and Skoog (MS) basal nutrients and vitamins (Murashige and Skoog 1962) fortified with 3 % (w/v) sucrose, adjusted to pH 5.8 using 1 N KOH and solidified with 0.8 % (w/v) plant agar (Duchefa Biochemie, The Netherlands). Thidiazuron (TDZ) was filter-sterilised and added to autoclaved medium. Other PGRs were added to MS medium prior to pH adjustment (5.8) and sterilisation (1.2 kg cm^−2^ for 20 min). Cultures were maintained at 25 ± 1 °C with a 16/8 h light/dark photoperiod at 45 µmol m^−2^ s^−1^ photosynthetic photon flux density provided by cool white fluorescent light (Philips 40 W tubes). Shoot tips (1.0–1.5 cm long) isolated from disinfected shoots were cultured on MS medium supplemented with 0, 1.0, 2.0, 4.0, or 8.0 µM 6-Benzyladenine (BA) or TDZ alone or in combination with 2.7 µM α-naphthaleneacetic acid (NAA). For liquid cultures, the regenerated shoots (1.5–2.0 cm long) were placed on a balloon-type bubble bioreactor (3l) containing 1.5 L of MS liquid medium fortified with 8.0 µM BA and 2.7 µM NAA, and the air volume was adjusted to a constant flow rate of 0.2 air volume per medium per min. The number of explants developing shoots and mean number of shoots were recorded after 45 days of culture.

### Rooting and acclimatisation

The regenerated shoots more than 2.0 cm long were excised from the multiple shoots and cultured on PGR-free MS medium for root induction. The frequency of root induction, mean number of roots and root length were recorded after 30 days of culture. Rooted plantlets were removed from culture medium and washed thoroughly with sterile distilled water. Plantlets were transplanted into plastic box containing peat moss, perlite and vermiculite (1:1:1, v/v/v), irrigated at 2 days’ interval with quarter-strength MS basal nutrients solution and maintained in the greenhouse (22 ± 5 °C, 70–80 % relative humidity). The survival rate of plantlets was recorded after 4 weeks.

### Extraction and quantification of carotenoids and tocopherols

For bioactive compound analysis, leaves were collected from greenhouse-grown plants (3-month-old) and regenerated shoots (30-day-old shoots grown in MS medium, MS + 2.0 µM BA or MS + 2.0 µM TDZ). Carotenoids and tocopherols were extracted and quantified according to Saini et al. (2012, 2014a) with some modifications. Under low light (5–8 µmol m^−2^ s^−1^), 2.0 g of each leaf sample was homogenized with 10 mL of chilled acetone containing 0.1 % (w/v) butylated hydroxytoluene (BHT), centrifuged at 5000*g* for 5 min at 4 °C and the supernatant collected. The extraction was repeated until the pellets became colourless. Supernatants were pooled (total volume 30–40 mL), vacuum-dried in a rotary evaporator (Büchi RE 111, Switzerland) at 35 °C, re-suspended in 5.0 mL of chilled acetone containing 0.1 % BHT and anhydrous sodium sulphate added (around 100 mg). The extract was filtered through a 0.45-µM Whatman syringe filter (PVDF filter media, Sigma Aldrich, St. Louis, MO, USA) into an amber high-performance liquid chromatography (HPLC) vial and analysed on the same day.

The analysis of carotenoids and tocopherols was carried out using an Agilent model 1100 HPLC (Agilent, Palo Alto, CA, USA) unit equipped with a YMC, C30 carotenoid column, 250 × 4.6 mm, 5 mm (YMC, Wilmington, NC). The column thermostat was maintained at 25 °C temperature. 20 μL of standards and samples was injected with auto sampler. The mobile phase consisted of 81:15:4 (v/v/v) methanol/methyl tertiary butyl ether (MTBE)/water (solvent A) and 91:9 (v/v) MTBE/methanol (solvent B). The gradient elution was 0–50 % B in 45 min followed by 0 % B in the next 5 min and 5 min post run at a flow rate of 1 mL/min. Carotenoids and tocopherols were detected at 450 and 295 nm, respectively. Quantitative determination of compounds was conducted by comparison with dose–response curves constructed from authentic standards of carotenoids and tocopherols. Authentic standards of carotenoid, all-E-lutein and all-E-zeaxanthin were purchased from Cayman Chemical Company, Michigan, USA. 9′-Z-neoxanthin, and all-E-violaxanthin was purchased from DHI LAB products Hoersholm, Denmark. α-carotene was purchased from Santa Cruz Biotechnology, Texas, USA. All-E-β-cryptoxanthin, all-E-β-carotene and tocopherol standards (δ, γ, and α-tocopherol) were purchased from Sigma Aldrich, St. Louis, MO, USA.

### Fatty acid extraction and fatty acid methyl esters (FAMEs) preparation

Total lipids were extracted according to Bligh and Dyer (1959) and Saini et al. (2014b) with minor modifications. Briefly, 2.0 g of each leaf sample was transferred into amber glass vial and homogenized with 20 mL chloroform and 10 mL methanol, centrifuged at 5000*g* for 5 min at 4 °C and supernatant collected. The extraction was repeated until the pellets became colourless. Supernatants were pooled (total volume 50–70 mL) in a 250-mL separating funnel and partitioned with 30 mL of 0.85 % (w/v) sodium chloride (NaCl). Lower organic (chloroform) phase was collected into pre weighted tube, completely dried in vacuum rotary evaporator and total lipid content determined gravimetrically. Fatty acid methyl esters (FAMEs) were prepared by conventional anhydrous methanolic HCl (Hydrochloric acid) method. Briefly, 4 mL of 5 % (v/v) anhydrous methanolic HCl was added to lipid sample in a graduated glass tube, fitted with refluxing tube and refluxed for 3 h at 60 °C in water bath. After cooling, FAMEs were washed sequentially with 5 % (w/v) NaCl followed by 2 % (w/v) sodium bicarbonate (NaHCO_3_) and recovered in 20 mL hexane. The hexane extract was dried up to 1 mL in vacuum rotary evaporator, transferred to 2 mL glass GC tubes, completely dried under nitrogen gas and stored at −20 °C in the presence of anhydrous sodium sulphate.

### Gas chromatography–mass spectrophotometry (GC–MS) analysis of FAMEs

FAMEs were analysed by GC-2010 Plus Gas chromatography (Shimadzu, Japan) equipped with AOC-20 i Auto injector and GCMS-QP2010 SE Gas chromatography mass spectrophotometry using a slightly polar RXi-5Sil column (Restek; 30 m × 250 μM id × 0.25 μM film). Injector port and detector were set up at 250 and 230 °C, respectively, and helium (He) was used as carrier gas. Initially, column temperature was maintained at 120 °C for 5 min, followed by increasing to 240 °C in 30 min and held at 240 °C for 25 min. The FAMEs were identified by comparing their fragmentation pattern and retention time (RT) with authentic standards and also with the NIST library (Saini et al. 2014b). Standard mixtures of fatty acid methyl esters (CRM47885–Supelco 37 Component FAME Mix) were purchased from Sigma Aldrich, St. Louis, MO, USA.

### Statistical analysis

For each treatment, 25 shoot tips, 50 shoots, or 100 plantlets were used and the experiment was repeated three times. All data were subjected to analysis of variance using SAS program (Release 9.2, SAS Institute, NC, USA). Differences between the mean values were assessed with Duncan’s multiple range test at *P* ≤ 0.05.

## Results and discussion

### Axillary shoot multiplication

**Fig. 1:**
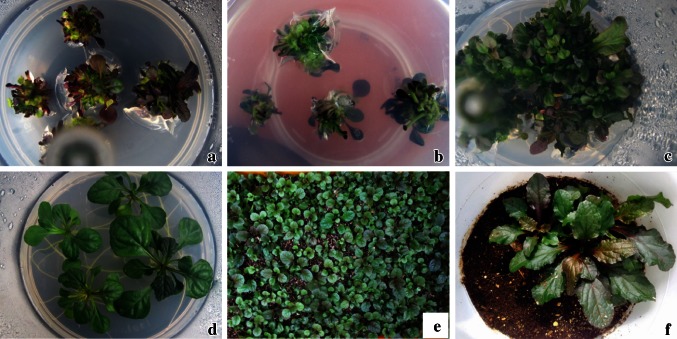
In vitro propagation of *A. multiflora*; **a**, **b** multiple shoots’ (*green* and *purple shoots*) induction on MS medium fortified with 8.0 µM BA; **c** shoot proliferation on MS medium fortified with 8.0 µM BA and 2.7 µM NAA; **d** rooting on MS basal medium; **e**, **f** acclimatised plants

In vitro propagation through axillary shoot proliferation is an efficient method for large-scale production of true-to-type planting material of important plants. Shoot tip explant is widely used for in vitro shoot proliferation of various plants such as *Cucumis sativus* (Sangeetha and Venkatachalam 2014), *Corchorus capsularis* (Saha et al. 2014) and *Pterocarpus santalinus* (Balaraju et al. 2011). Shoot tip explants cultured on PGRs-free MS medium produced a mean of 1.3 shoots per explant, and the frequency of shoot induction was 46.1 %. Shoot proliferation (>2 shoots) was achieved when the shoot tip explants were cultured on MS medium fortified with BA or TDZ individually or in combination with NAA (Table 1). Sivanesan and Park (2015) reported that addition of PGRs was required for shoot proliferation from nodal explants of *A. multiflora*. The concentration and ratio of PGRs often determine the morphogenetic response of the explant. Shoot tip explants remained green and the regenerated shoots developed roots on PGR-free MS medium, whereas the explants changed into purple or red colour and they produced multiple shoots (both green and pigmented shoots) when MS medium was fortified with BA or TDZ (Fig. 1a, b). Anthocyanins are pigmented flavonoids that are responsible for most of the red, pink, purple and blue colours found in plants (Deikman and Hammer 1995). Anthocyanin biosynthesis is influenced by chemical and physical factors. Cytokinins have been shown to enhance anthocyanin accumulation in in vitro cultures of many plants including *Ajuga* (Callebaut et al. 1990, 1997; Deikman and Hammer 1995; Ji et al. 2015). The frequency of shoot induction and the average number of shoots produced per shoot tip explant increased with increasing concentrations of BA in MS basal medium. Maximum frequency of shoot formation (78.3 %), with a mean of 9.3 shoots per explant was obtained on MS medium fortified with 8.0 µM BA. The positive effect of BA on shoot formation has also been reported in various plants such as *Ajuga reptans* (Preece and Huetteman 1994), ginger (Das et al. 2013), *Moringa oleifera* (Saini et al. 2012) and *Scrophularia takesimensis* (Jeong and Sivanesan 2015). Of the various concentrations of TDZ studied, maximum number of shoots (8.5) was obtained on MS medium fortified with 2.0 µM TDZ. The average number of shoots induced per explant decreased with concentrations of TDZ above 2.0 µM (Table 1). Similarly, the inhibitory effects of high concentrations of TDZ (4.0–16 µM TDZ) on adventitious shoot formation has been reported in *A. multiflora* (Sivanesan et al. 2011).

**Table 1:** Effect of PGRs on multiple shoot formation from shoot tip explants of *A. multiflora*

PGRs (µM)			Explants responding with shoots (%)	Number of shoots per explant
BA	TDZ	NAA		
0.0	0.0	0.0	46.1 ± 3.2k	1.3 ± 0.5k
1.0	0.0	0.0	54.5 ± 2.6j	2.4 ± 1.2kj
2.0	0.0	0.0	67.5 ± 2.6i	5.3 ± 1.5gh
4.0	0.0	0.0	71.9 ± 3.9g	6.6 ± 1.5fg
8.0	0.0	0.0	78.3 ± 2.0f	9.3 ± 1.6d
1.0	0.0	2.7	85.4 ± 3.1d	4.9 ± 1.1hi
2.0	0.0	2.7	97.8 ± 1.7a	11.8 ± 2.1c
4.0	0.0	2.7	100 ± 0.0a	13.6 ± 2.3b
8.0	0.0	2.7	100 ± 0.0a	17.1 ± 2.6a
0.0	1.0	0.0	73.1 ± 2.2g	3.5 ± 1.1ij
0.0	2.0	0.0	82.9 ± 3.4e	8.5 ± 0.9de
0.0	4.0	0.0	90.3 ± 2.5c	5.1 ± 0.8gh
0.0	8.0	0.0	94.3 ± 2.1b	4.6 ± 0.7ih
0.0	1.0	2.7	95.4 ± 1.7b	6.6 ± 1.1fg
0.0	2.0	2.7	100 ± 0.0a	12.1 ± 1.8c
0.0	4.0	2.7	100 ± 0.0a	7.5 ± 1.1def
0.0	8.0	2.7	100 ± 0.0a	6.0 ± 1.4fgh

Mean ± SD within a column followed by the same letters are not significantly different (*P* ≤ 0.05)

**Fig. 2:**
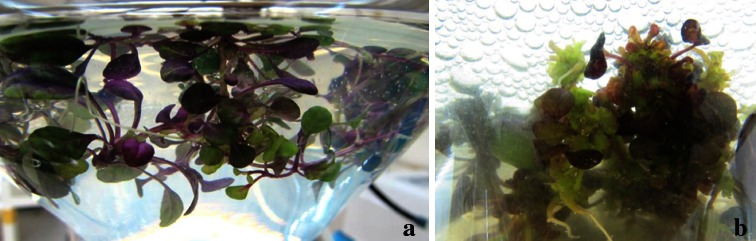
Shoot proliferation in a balloon-type bubble bioreactor containing MS liquid medium fortified with 8.0 µM BA and 2.7 µM NAA. **a** After 7 days of culture; **b** after 28 days of culture

**Fig. 3:**
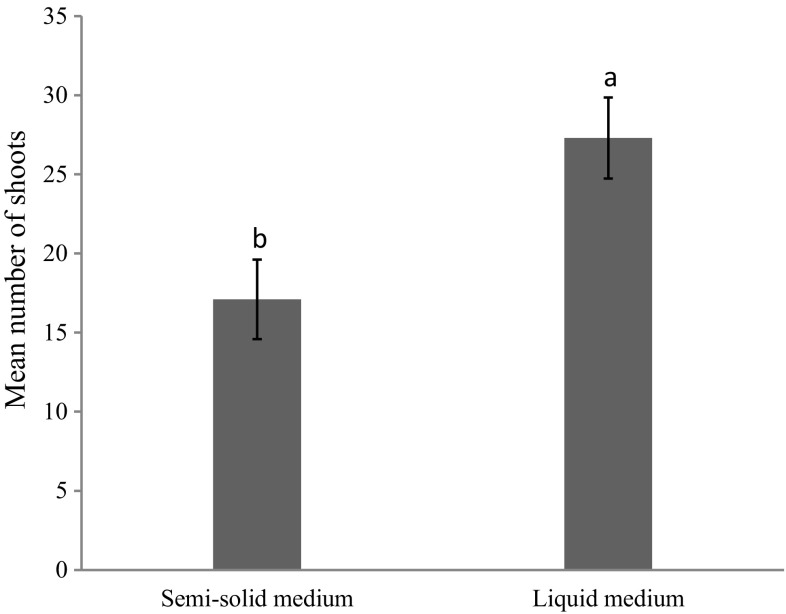
Effect of liquid and semi-solid culture media on shoot proliferation of *A. multiflora* after 45 days of culture

In a previous study, BA combination with NAA (2.7 µM) was found best for shoot proliferation from nodal explants of *A. multiflora* as compared to BA in combination with IAA or IBA (Sivanesan and Park 2015). Addition of BA or TDZ in combination with 2.7 µM NAA markedly enhanced shoot proliferation. The highest number of shoots (17.1) was achieved when 8.0 µM BA was combined with 2.7 µM NAA (Table 1; Fig. 1c). Similar results have also been reported in *Ajuga bracteosa* (Kaul et al. 2013), *Senecio cruentus* (Sivanesan and Jeong 2012) and *Sida cordifolia* (Sivanesan and Jeong 2007). Liquid culture has proven successful in large-scale commercial propagation of plants. The shoot tip explants inoculated in a balloon-type bubble bioreactor containing MS liquid medium with 8.0 μM BA and 2.7 μM NAA synthesised purple pigments within a week of culture (Fig. 2a), and the pigment expression may due to chemical or physical stress in liquid culture conditions. Callebaut et al. (1990, 1997) reported the pigment accumulation in callus and cell suspension cultures of *A. reptans*. The explants developed multiple shoots within 4 weeks of culture (Fig. 2b). The average number of shoots increased 1.6-fold when the shoot tips were cultured in liquid medium as compared with semi-solid medium (Fig. 3). Improved shoot proliferation in liquid systems has also been reported in several plants and this may be due to large surface area, better nutrient and water uptake (Pati et al. 2011; Savio et al. 2012).

### Rooting and acclimatisation

The successful rooting of regenerated shoots and acclimatisation of in vitro-developed plantlets to the greenhouse or field conditions are the most important steps in micropropagation. Root induction is often inhibited by the cytokinins used to induce shoot multiplication. Auxins play an important role on adventitious root formation (Pacurar et al. 2014). Thus, auxins have been used to stimulate in vitro rooting of several plants (Saha et al. 2014; Sangeetha and Venkatachalam 2014; Jeong and Sivanesan 2015). Kaul et al. (2013) reported root formation from excised shoots of *A. bracteosa* required auxin supplement in MS basal medium. However, in this study, the regenerated shoots developed roots on PGRs-free MS basal medium after 7 days of culture and roots having well-developed secondary branches after 30 days of culture (Fig. 1d). On this medium, 100 % of shoots rooted with a mean number of 7.2 ± 1.6 roots per shoot. Similar result has also been reported in *A. multiflora* (Sivanesan et al. 2011; Sivanesan and Jeong 2014) and *A. reptans* (Preece and Huetteman 1994). The in vitro-developed plantlets were successfully acclimatised in the greenhouse with 100 % of survival (Fig. 1e, f). The acclimatised plants grew well and did not show any variation in morphology when compared with donor plant.

### Contents of carotenoids and tocopherol

**Fig. 4:**
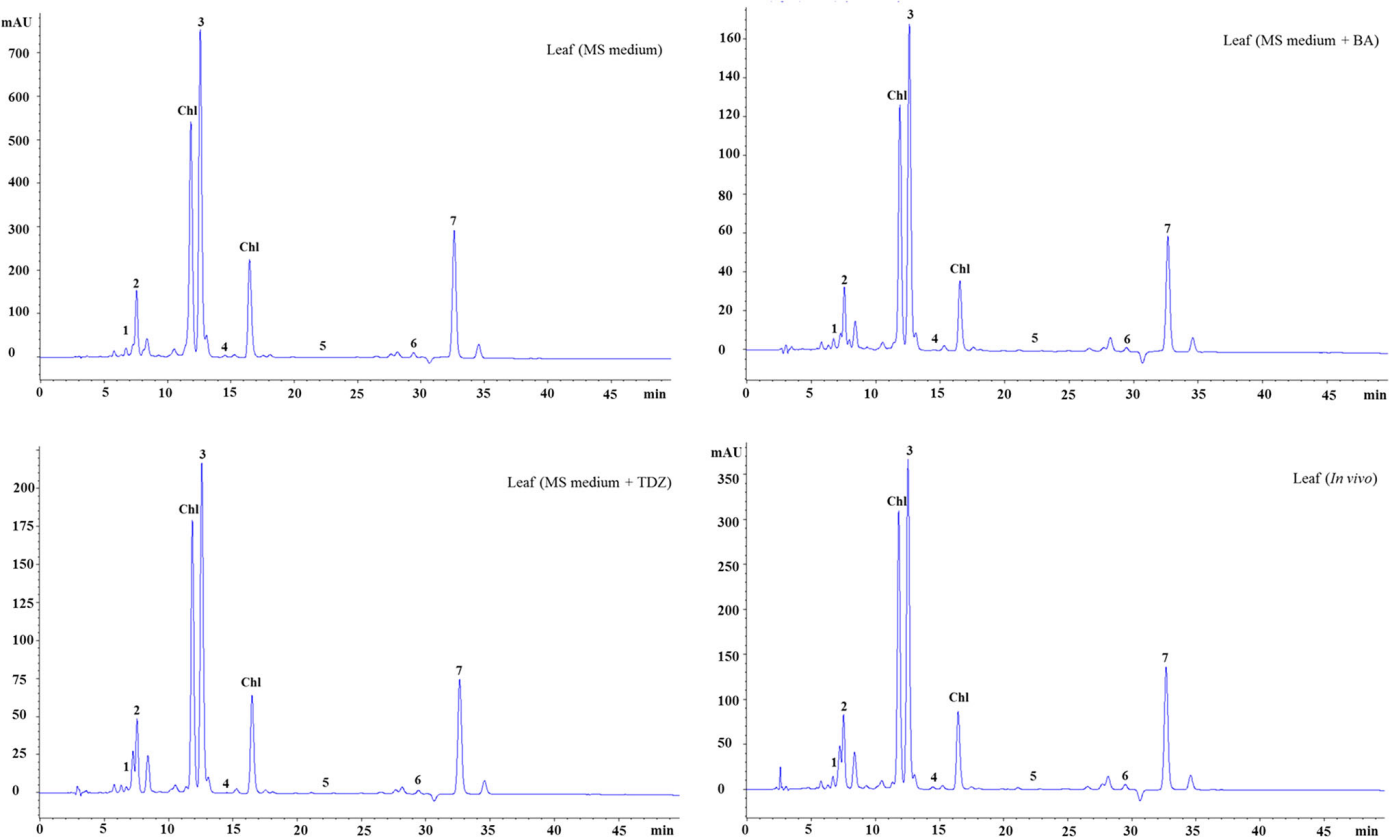
HPLC chromatograms (UV, 450 nm) of carotenoids in leaf tissues of *A. multiflora*. *1* all-E-violaxanthin (RT: 6.6); *2* 9′-Z-neoxanthin (RT: 7.5); *3* all-E-lutein (RT: 12.5); *4* all-E-zeaxanthin (RT: 14.5); *5* all-E-β-cryptoxanthin (RT: 22.7); *6* α-carotene (RT: 29.3); and *7* All-E-β-carotene (RT: 32.5); Chl chlorophyll

Carotenoids and tocopherols are mainly found in green leafy tissue of plants. Several studies have shown that carotenoids and tocopherols can play an important role in the prevention of skin damage, cancer, cardiovascular, eye and neurodegenerative diseases (Fraser and Bramley 2004; Fiedor and Burda 2014; Esteban et al. 2015). Concentrations of individual and total carotenoids in *A. multiflora* leaves are shown in Table 2. The total carotenoid content (TCC) varied from 150.0 to 662.31 μg g^−1^ FW in leaves. It is worth mentioning that TCC in leaves of *A. multiflora* is higher than several leafy vegetables such as amaranth (78.99 mg 100 g^−1^ DW), broccoli (0.1 mg g^−1^ FW), cabbage (0.03 mg g^−1^ FW), chard (0.19 mg g^−1^ FW), chicory (3.94 mg 100 g^−1^ FW), dandelion (6.34 mg 100 g^−1^ FW), garden rocket (8.24 mg 100 g^−1^ FW), lettuce (8.48 mg 100 g^−1^ FW), spinach (0.2 mg g^−1^ FW) and wild rocket (7.18 mg 100 g^−1^ FW) previously reported (Muller 1997; Raju et al. 2007; Znidarcic et al. 2011; Mitic et al. 2013). Figure 4 shows a HPLC chromatogram of carotenoids detected in *A. multiflora* leaves. The most abundant carotenoid in *A. multiflora* leaves was all-E-lutein (89.4–382.6 μg g^−1^ FW) followed by all-E-β-carotene (32.0–156.7 μg g^−1^ FW), 9′-Z-neoxanthin (14.2–63.4 μg g^−1^ FW), all-E-violaxanthin (13.0–45.9 μg g^−1^ FW), all-E-zeaxanthin (1.3–2.5 μg g^−1^ FW) and all-E-β-cryptoxanthin (0.3–0.9 μg g^−1^ FW). All-E-zeaxanthin and all-E-cryptoxanthin were not detected in leaves of in vitro-regenerated shoots cultured on MS medium with 2.0 µM BA or TDZ. The highest TCC was found in leaves of in vitro-regenerated shoots (662.31 μg g^−1^ FW) cultured on MS basal medium as compared with other leaf samples (Table 2). The TCC was significantly decreased in leaves of in vitro-regenerated shoots cultured on MS medium with 2.0 µM BA or TDZ. The inhibitory effect of cytokinin on TCC has been reported in banana (Aremu et al. 2012) and *Lallemantia iberica* (Pourebad et al. 2015).

**Table 2:** Carotenoid content and composition in leaf tissues of *A. multiflora*

Plant materials	All-E-violaxanthin	9′-Z-neoxanthin	All-E-lutein	All-E-zeaxanthin	All-E-β-cryptoxanthin	α-Carotene	All-E-β-carotene	Total carotenoids
Leaf (MS)	45.90 ± 2.20a	63.48 ± 2.31a	382.63 ± 8.8a	2.49 ± 069a	0.93 ± 0.10a	10.11 ± 1.2a	156.78 ± 5.56a	662.31a
Leaf (MS + BA)	13.04 ± 0.71d	14.22 ± 0.49d	89.40 ± 4.4d	nd	nd	1.35 ± 0.55c	32.00 ± 1.21d	150.00d
Leaf(MS + TDZ)	17.74 ± 0.73c	17.72 ± 0.62c	107.38 ± 5.1c	nd	nd	3.64 ± 0.67b	35.52 ± 1.22c	182.01c
Leaf (in vivo)	20.70 ± 0.74b	34.09 ± 0.59b	190.53 ± 5.5b	1.31 ± 0.12b	0.30 ± 0.05b	3.57 ± 0.71b	71.18 ± 0.59b	321.67b

Values (μg g^−1^ FW) are mean of triplicates’ determination

Different letters indicate statistically significant differences between the means (*P* < 0.05)

*nd* not detected

**Fig. 5:**
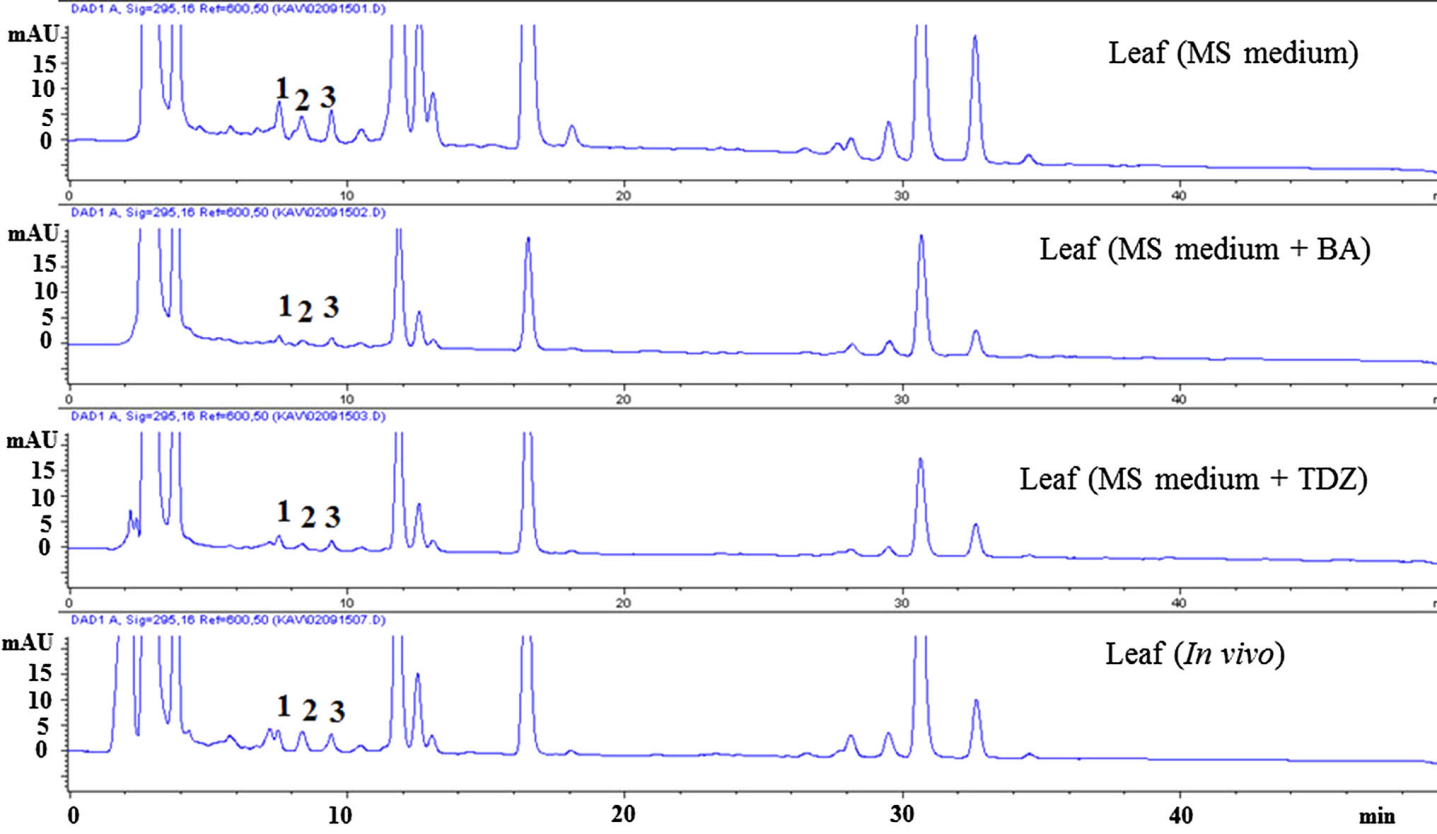
HPLC chromatograms (UV, 295 nm) of tocopherols in leaf tissues of *Ajuga multiflora*. *1* δ-tocopherol (RT: 7.3); *2* γ-tocopherol (RT: 8.3); *3* α-tocopherol (RT: 9.4)

Figure 5 shows a HPLC chromatogram of tocopherols detected in *A. multiflora* leaf extracts. The total content of tocopherols ranged from 68.9 to 253.6 μg g^−1^ FW in leaf samples, which was greater than that in many fruits (1.1–84 μg g^−1^ FW), vegetables (1.0–30 μg g^−1^ FW), legumes (4.8–16.7 μg g^−1^ FW) and cereals (17–60 μg g^−1^ FW) reported earlier (Caretto et al. 2010). The highest level of total tocopherols was measured in leaves of in vitro-regenerated shoots cultured on MS basal medium (253.6 μg g^−1^ FW), followed by leaves of greenhouse-grown plants (187.2 μg g^−1^ FW) and leaves of in vitro-regenerated shoots cultured on MS basal medium with 2.0 µM BA (68.9 μg g^−1^ FW) or TDZ (68.8 μg g^−1^ FW). The most abundant tocopherol in *A. multiflora* leaves was α-tocopherol followed by γ- and δ-tocopherol (Table 3). α-Tocopherol is reported to have greater vitamin E activity and is largely present in leaf tissues of various plant species (Carvalho et al. 2013). The content of α-tocopherol in *A. multiflora* leaves (101.1 μg g^−1^ FW) was higher than the value reported in *Amaranthus caudatus* (11.3 μg g^−1^ FW), *Arabidopsis* (10 μg g^−1^ FW), *Chenopodium quinoa* (1.98 μg g^−1^ FW), sunflower (14 μg g^−1^ FW) and tobacco (43 μg g^−1^ FW) cell cultures (Gala et al. 2005; Antognoni et al. 2008; Harish et al. 2013) while lower than the value reported in *Carthamus tinctorius* (167.7 μg g^−1^ FW) and *Vitis vinifera* (261.5 μg g^−1^ FW) cell cultures (Chavan et al. 2011; Cetin 2014).

**Table 3:** Tocopherol content in leaf tissues of *A. multiflora*

Plant materials	δ-Tocopherol	γ-Tocopherol	α-Tocopherol	Total tocopherols
Leaf (MS)	65.27 ± 2.74a	87.21 ± 2.21a	101.09 ± 4.91a	253.57a
Leaf (MS + BA)	18.74 ± 1.23c	22.29 ± 1.81c	27.85 ± 2.86c	68.89c
Leaf (MS + TDZ)	25.94 ± 1.31b	17.41 ± 1.21d	25.46 ± 2.11c	68.81c
Leaf (in vivo)	66.91 ± 3.12a	70.32 ± 2.38b	49.97 ± 2.31b	187.21b

Values (μg g^−1^ FW) are mean of triplicates’ determination

Different letters indicate statistically significant differences between the means (*P* < 0.05)

### Composition of fatty acids

Fatty acids and their derivatives are used in pharmaceutical and food industries. The composition of fatty acids in leaf samples was analysed by GC–MS (Fig. S1). Table 4 shows the fatty acid composition of *A. multiflora* leaf samples. Significant differences in fatty acid content were observed among leaf samples. Linolenic acid (49.03–52.59 %) was detected in higher amounts in *A. multiflora* leaf samples followed by linoleic acid (18.95–21.39 %) and palmitic acid (15.79–18.66 %). Several studies have shown that linolenic acid may play an important role in the prevention of cardiovascular disease (Pan et al. 2012). The amounts of capric acid, lauric acid, myristic acid, pentadecylic acid, palmitic acid, heptadecenoic acid, margaric acid, oleic acid, stearic acid, nonadecylic acid, gadoleic acid, arachidic acid, heneicosylic acid, erucic acid, behenic acid, tricosylic acid and lignoceric acid were lower (Table 4). Leaves of in vitro regenerated shoots cultured on MS basal medium did not contain capric, heptadecenoic or nonadecylic acids. The leaf samples contained 21.71–28.68 % saturated fatty acids, 3.14–4.62 % monounsaturated fatty acids and 67.98–73.67 % polyunsaturated fatty acids. Halder and Gadgil (1984) reported that the proportion of polyunsaturated fatty acids in callus cultures of *Cucumis melo* was greater than the saturated fatty acids.

**Table 4:** Composition of fatty acids in leaf tissues of *A. multiflora*

S. no	Fatty acids	RT	Leaf (MS)	Leaf (MS + BA)	Leaf (MS + TDZ)	Leaf (in vivo)
1	Capric acid (C10:0)	7.77	nd	0.06a	0.05a	0.03b
2	Lauric acid (C12:0)	13.53	0.11b	0.24a	0.21a	0.19a
3	Myristic acid (C14:0)	19.22	0.40a	0.41a	0.42a	0.38a
4	Pentadecylic acid (15:0)	21.88	0.25a	0.26a	0.25a	0.26a
5	Palmitoleic acid (C16:1, cis-9)	23.89	0.18c	0.22c	0.41a	0.25b
6	Palmitic acid (C16:0)	24.56	15.79b	18.47a	18.66a	18.04a
7	Heptadecenoic acid (17:1, cis-10)	26.21	nd	nd	0.07b	0.16a
8	Margaric acid (17:0)	26.84	0.42c	0.52b	0.59a	0.51b
9	Linoleic acid (C18:2, cis-9,12)	28.49	21.09a	19.26a	18.95a	21.39a
10	Linolenic acid (C18:3, cis-9,12,15)	28.81	52.59a	49.63b	49.03b	51.43a
11	Oleic acid (C18:1, cis-9)	28.85	4.01a	3.03b	2.43c	2.35c
12	Stearic acid (C18:0)	29.22	3.45c	4.12b	5.58a	3.14c
13	Nonadecylic acid (19:0)	31.35	nd	0.05a	0.07a	0.04a
14	Gadoleic acid (20:1, cis-11)	32.93	0.02b	0.04a	0.04a	0.06a
15	Arachidic acid (C20:0)	33.48	0.36b	0.88a	0.84a	0.39b
16	Heneicosylic acid (21:0)	35.52	0.24a	0.24a	0.24a	0.15b
17	Erucic acid (C22:1, cis-13)	37.26	0.40a	0.28b	0.39a	0.31b
18	Behenic acid (C22:0)	37.97	0.27b	0.59a	0.63a	0.30b
19	Tricosylic acid (23:0)	40.84	0.15d	0.93a	0.33b	0.23c
20	Lignoceric acid (C24:0)	44.63	0.26c	0.75a	0.80a	0.39b
	Total saturated fatty acids (SFA)		21.71c	27.53a	28.68a	24.05b
	Total monounsaturated fatty acids (MUFA)		4.62a	3.57b	3.34b	3.14b
	Total polyunsaturated fatty acids (PUFA)		73.67a	68.90b	67.98b	72.82a
	PUFA: SFA		3.39a	2.50b	2.37b	3.03a
	PUFA: MUFA		15.95d	19.28c	20.35b	23.20a
	Total lipids		3.51a	3.32b	3.35b	3.40b

Values are percentages of the total fatty acids, from an average of triplicate extractions and analyses

Different superscript letters indicate statistically significant differences among different explants (between the columns) (*P* < 0.05)

*nd* not detected

In conclusion, an improved in vitro propagation protocol has been developed for *A. multiflora*. The effect of culture media on composition of carotenoid, tocopherol and fatty acids in leaf tissues of *A. multiflora* is reported for the first time. The culture media had a significant effect on the production of bioactive compounds. The MS basal medium was more effective than MS medium with BA or TDZ for the production of bioactive compounds. The contents of carotenoids, tocopherols and polyunsaturated fatty acids were higher in green leaves of in vitro-regenerated shoots than purple-green leaves of in vitro-regenerated shoots or greenhouse-grown plants. This protocol can be useful for large-scale production of bioactive compounds of *Ajuga* species.

## Electronic supplementary material

Below is the link to the electronic supplementary material.

Supplementary material 1 (DOCX 399 kb)
