# Supplementary material for: In vitro propagation, carotenoid, fatty acid and tocopherol content of Ajuga multiflora Bunge
Source: 3 Biotech. 2016 Mar 14;6(1):91. doi: 10.1007/s13205-016-0376-z (PMC4791420; doi:10.1007/s13205-016-0376-z)
Supplement: Supplementary file 1 — Supplementary material 1 (DOCX 399 kb) [file 13205_2016_376_MOESM1_ESM.docx]

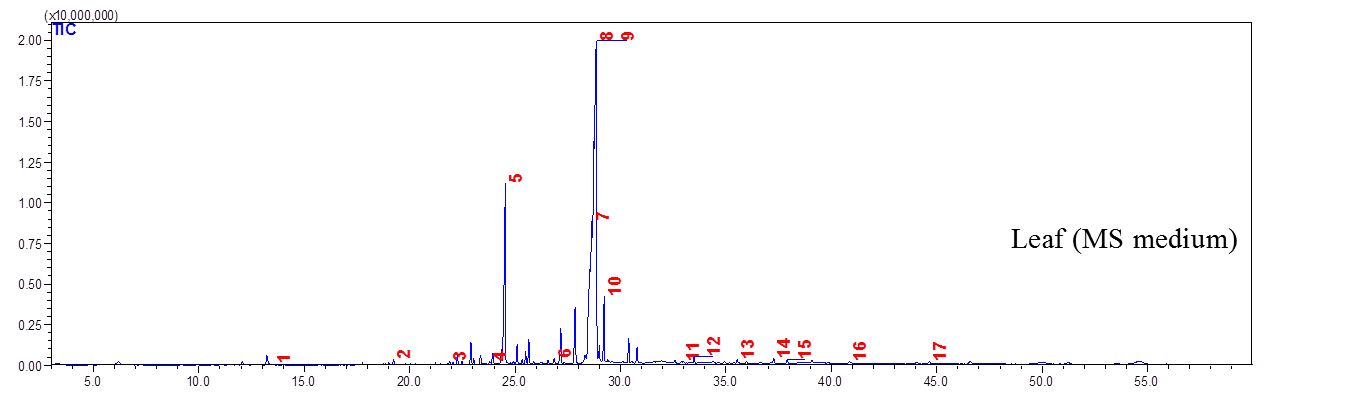


Figure S1. GC chromatograms of fatty acid methyl esters. 1 lauric acid (C12:0); 2 myristic acid (C14:0); 3 pentadecylic acid (15:0); 4 palmitoleic acid (C16:1, cis-9); 5 palmitic acid (C16:0); 6 margaric acid (17:0); 7 linoleic acid (C18:2, cis-9,12); 8 linolenic acid (C18:3, cis-9,12,15); 9 oleic acid (C18:1, cis-9); 10 stearic acid (C18:0); 11 gadoleic acid (20:1, cis-11); 12 arachidic acid (C20:0); 13 heneicosylic acid (21:0); 14 erucic acid (C22:1, cis-13); 15 behenic acid (C22:0); 16 tricosylic acid (23:0); 17 lignoceric acid (C24:0)


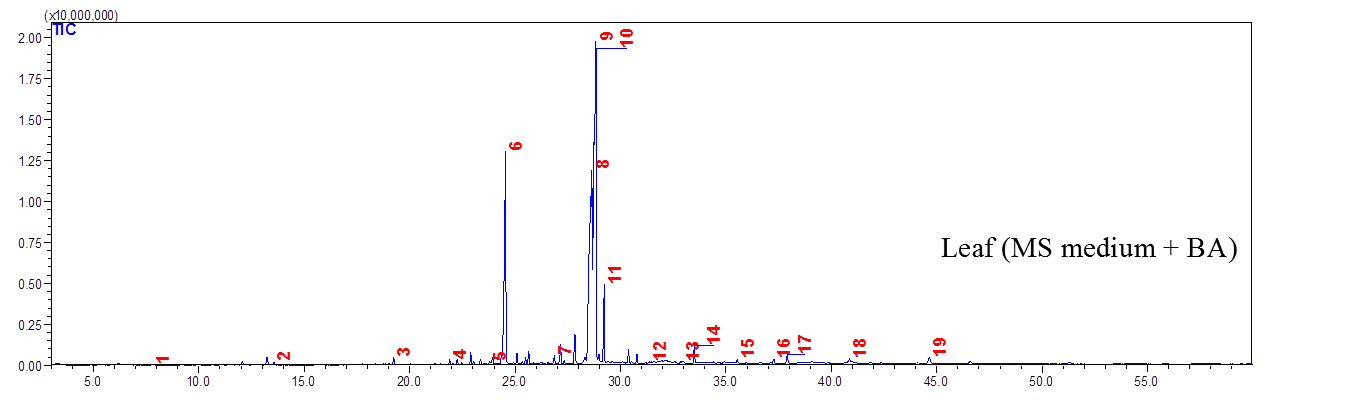


Figure S1. GC chromatograms of fatty acid methyl esters. 1 capric acid (C10:0); 2 lauric acid (C12:0); 3 myristic acid (C14:0); 4 pentadecylic acid (15:0); 5 palmitoleic acid (C16:1, cis-9); 6 palmitic acid (C16:0); 7 margaric acid (17:0); 8 linoleic acid (C18:2, cis-9,12); 9 linolenic acid (C18:3, cis-9,12,15); 10 oleic acid (C18:1, cis-9); 11 stearic acid (C18:0); 12 nonadecylic acid (19:0); 13 gadoleic acid (20:1, cis-11); 14 arachidic acid (C20:0); 15 heneicosylic acid (21:0); 16 erucic acid (C22:1, cis-13); 17 behenic acid (C22:0); 18 tricosylic acid (23:0); 19 lignoceric acid (C24:0)


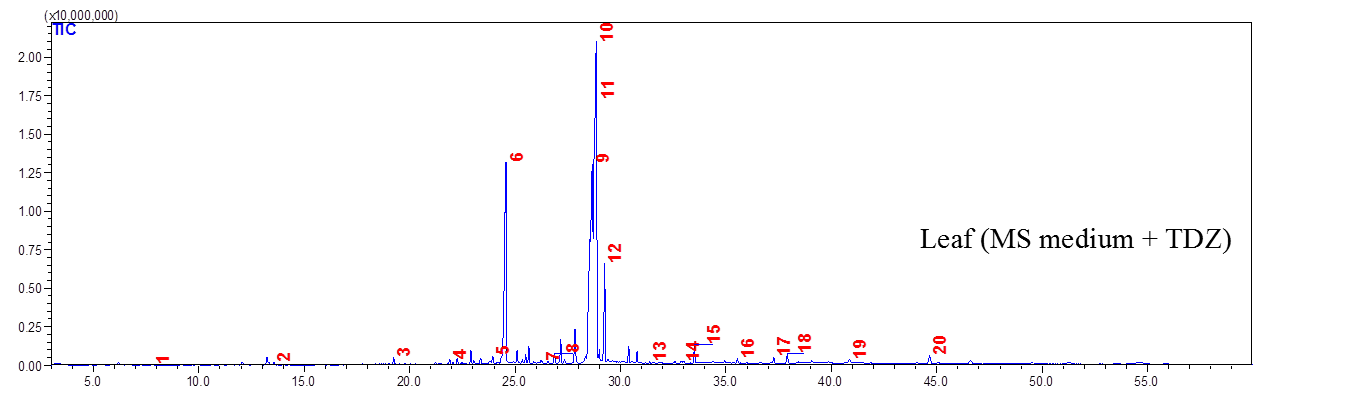


Figure S1. GC chromatograms of fatty acid methyl esters. 1 capric acid (C10:0); 2 lauric acid (C12:0); 3 myristic acid (C14:0); 4 pentadecylic acid (15:0); 5 palmitoleic acid (C16:1, cis-9); 6 palmitic acid (C16:0); 7 heptadecenoic acid (17:1, cis-10); 8 margaric acid (17:0); 9 linoleic acid (C18:2, cis-9,12); 10 linolenic acid (C18:3, cis-9,12,15); 11 oleic acid (C18:1, cis-9); 12 stearic acid (C18:0); 13 nonadecylic acid (19:0); 14 gadoleic acid (20:1, cis-11); 15 arachidic acid (C20:0); 16 heneicosylic acid (21:0); 17 erucic acid (C22:1, cis-13); 18 behenic acid (C22:0); 19 tricosylic acid (23:0); 20 lignoceric acid (C24:0)


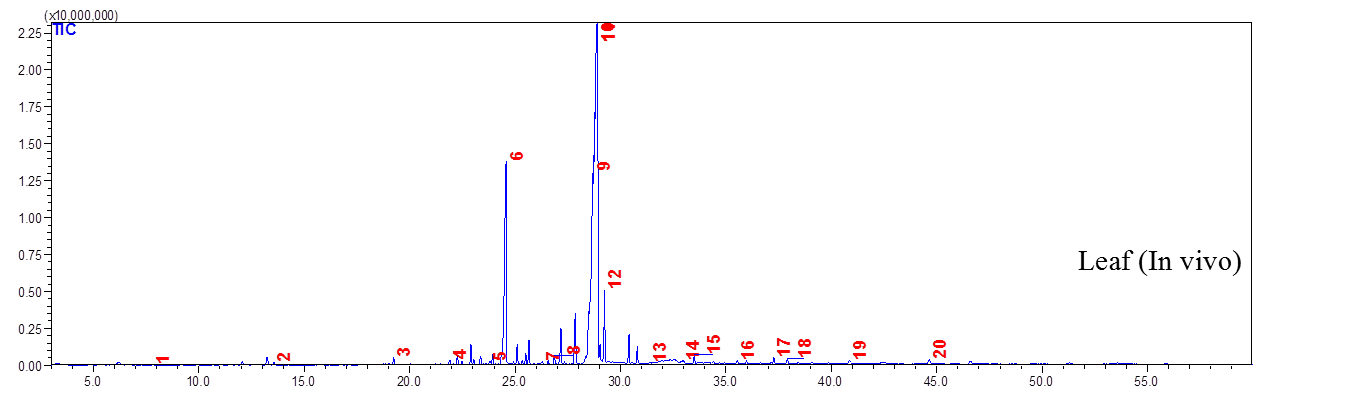


Figure S1. GC chromatograms of fatty acid methyl esters. 1 capric acid (C10:0); 2 lauric acid (C12:0); 3 myristic acid (C14:0); 4 pentadecylic acid (15:0); 5 palmitoleic acid (C16:1, cis-9); 6 palmitic acid (C16:0); 7 heptadecenoic acid (17:1, cis-10); 8 margaric acid (17:0); 9 linoleic acid (C18:2, cis-9,12); 10 linolenic acid (C18:3, cis-9,12,15); 11 oleic acid (C18:1, cis-9); 12 stearic acid (C18:0); 13 nonadecylic acid (19:0); 14 gadoleic acid (20:1, cis-11); 15 arachidic acid (C20:0); 16 heneicosylic acid (21:0); 17 erucic acid (C22:1, cis-13); 18 behenic acid (C22:0); 19 tricosylic acid (23:0); 20 lignoceric acid (C24:0)


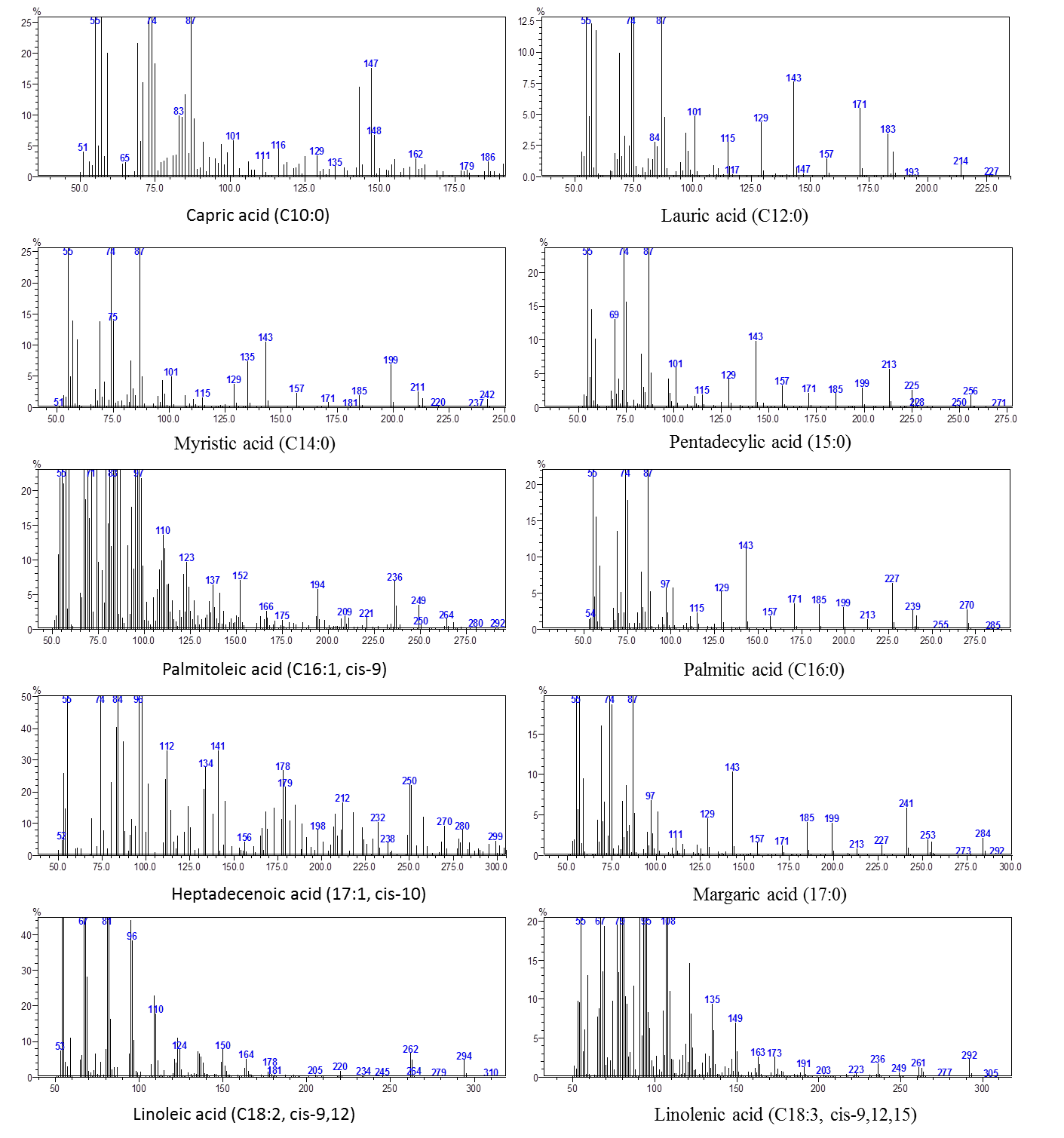


Figure S1. GC-MS spectrum of fatty acid methyl esters from *Ajuga multiflora* leaf sample


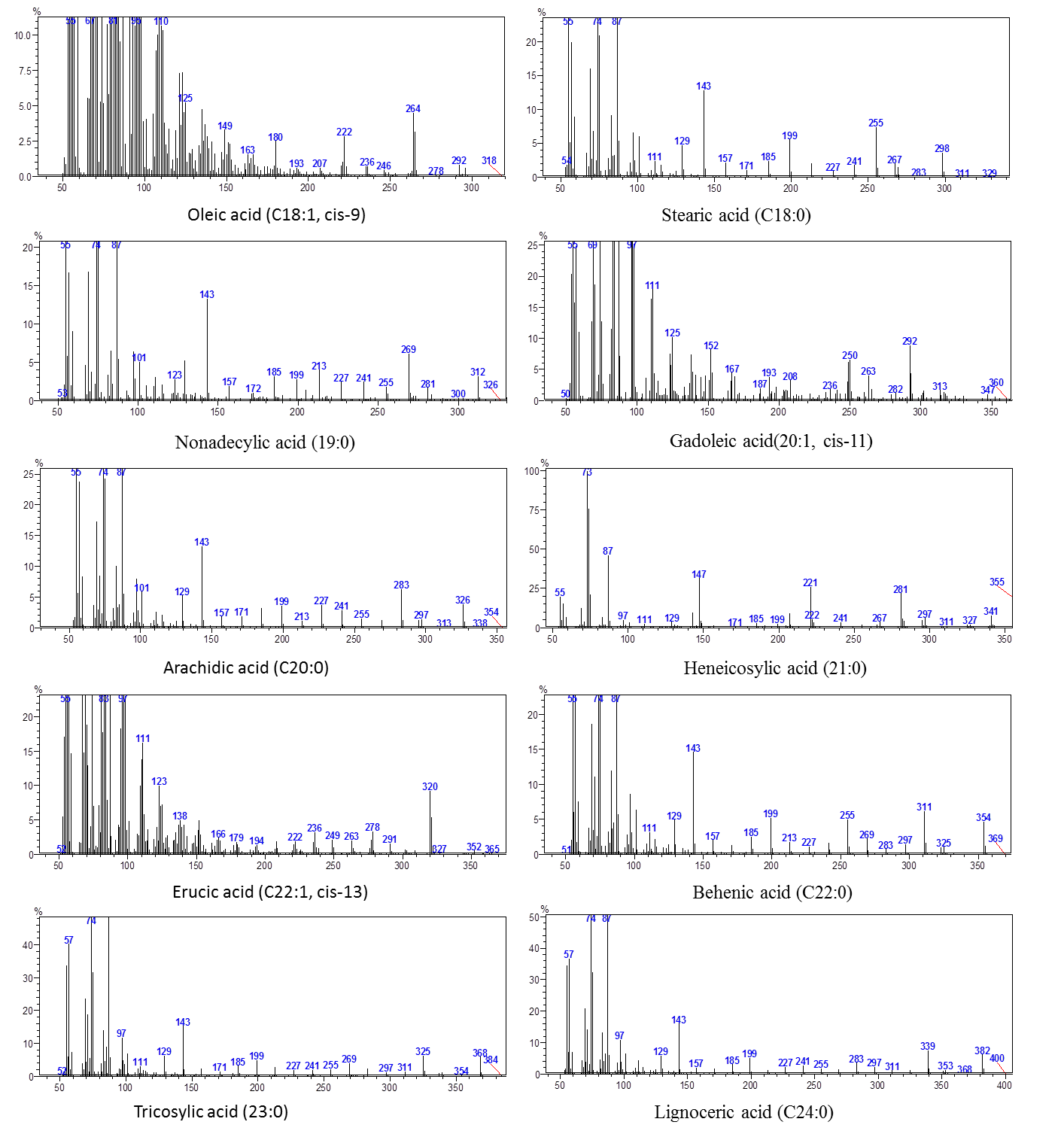


Figure S1. GC-MS spectrum of fatty acid methyl esters from *Ajuga multiflora* leaf sample
